# *Legionella longbeachae* wound infection: case report and review of reported *Legionella* wound infections

**DOI:** 10.3389/fcimb.2023.1178130

**Published:** 2023-04-26

**Authors:** Drifa Frostadottir, Lisa Wasserstrom, Karolin Lundén, Lars B. Dahlin

**Affiliations:** ^1^ Department of Translational Medicine – Hand Surgery, Lund University, Malmö, Sweden; ^2^ Department of Hand Surgery, Skåne University Hospital, Malmö, Sweden; ^3^ Clinical Microbiology, Laboratory Medicine Skåne, Lund, Sweden; ^4^ ESCMID Study Group for Legionella Infections (ESGLI), Basel, Switzerland; ^5^ Department of Biomedical and Clinical Sciences, Linköping University, Linköping, Sweden

**Keywords:** legionella, bacteria, *Legionella longbeachae*, cutaneous, infection, extrapulmonary manifestation

## Abstract

Extrapulmonary manifestations of infection with *Legionella* species, of which 24 may cause disease in humans, are very rare. Here, we describe a case of a 61-year-old woman with no history of immunosuppression presenting with pain and swelling of her index finger after a prick by rose thorns during gardening. Clinical examination showed fusiform swelling of the finger with mild redness, warmth, and fever. The blood sample revealed a normal white blood cell count and a slight increase in C-reactive protein. Intraoperative observation showed extensive infectious destruction of the tendon sheath, while the flexor tendons were spared. Conventional cultures were negative, while 16S rRNA PCR analysis identified *Legionella longbeachae* that also could be isolated on buffered charcoal yeast extract media. The patient was treated with oral levofloxacin for 13 days, and the infection healed quickly. The present case report, with a review of the literature, indicates that *Legionella* species wound infections may be underdiagnosed due to the requirement for specific media and diagnostic methods. It emphasizes the need for heightened awareness of these infections during history taking and clinical examination of patients presenting with cutaneous infections.

## Introduction

*Legionella* species (spp.) are gram-negative rod bacteria and present primarily in aqueous environments and soil, where they are adapted to survive and replicate intracellularly in protozoa. The intracellular mechanism behind *Legionella* infections is well described for *Legionella pneumophila* in mammalian tissue culture models, mainly using macrophage-like tissue, but also epithelial cells, where the primary mechanism of infection after internalization in these cell types is similar. Both *L. pneumophila* and *Legionella longbeachae* replicate inside the host by forming a *Legionella*-containing vacuole (LCV) that fuses with mitochondria and associates with the endoplasmic reticulum (ER) exit vesicle. By mimicking the host’s own ER vesicles, the bacteria can evade intracellular defenses, such as phagocytosis (Newton et al., 2010). The LCV delivers virulence-associated proteins by a Dot/Icm type 4B secretion system that modulates many different host signaling pathways, including anti-apoptotic effectors to prevent cell death during replication and proapoptotic effectors to promote exit of the bacteria at the end of the infection cycle (Mondino et al., 2020). Little is still known about the virulence of *L. longbeachae*, but studies have shown that mice are more susceptible to *L. longbeachae* than *L. pneumophila*, possibly due to the absence of flagella, which has a role in triggering cell death. Also, the presence of a capsule might protect *L. longbeachae* against phagocytosis (Cazalet et al., 2010).

There are over 60 *Legionella* species reported of which 24 may cause an infection in humans (Bell et al., 2021; Girolamini et al., 2022). The clinical manifestations of legionellosis vary from an acute, febrile illness (i.e., Pontiac fever) to potentially fatal pneumonia (i.e., Legionnaires’ disease). The mortality rate of Legionnaires’ disease varies and depends on the underlying status of the patient as well as the promptness of diagnosis and treatment. In Europe, *L. pneumophila* is the predominant pathogenic species causing 95% of Legionnaires’ disease. It spreads *via* amoeba present in man-made water systems, such as fountains, bubble baths, and cooling towers (Fields et al., 2002). Out of the remaining 5%, several other species can cause the disease with *L. longbeachae*, first isolated in 1980 in a patient with pneumonia in Long Beach, CA, USA (Whiley H, 2011), being the most common (Whiley H, 2011). However, due to a diagnostic shortage in detecting non-pneumophila species, these species are most likely underdiagnosed. Interestingly, in Australia and New Zealand, Legionnaires’ disease caused by *L. longbeachae* is as common as *L. pneumophila*, and since spreading has only been associated with compost and potting soil, commercially bagged soiled is marked with health warning symbols in these countries (Montanaro-Punzengruber et al., 1999). Extrapulmonary manifestations of *Legionella* spp. are extremely rare, but reported sites include brain (Charles et al., 2013), septic arthritis (Bemer et al., 2002; Linscott et al., 2004; Naito et al., 2007; Flendrie et al., 2011; Just et al., 2012; Thurneysen and Boggian, 2014; Banderet et al., 2017), pericarditis (Svendsen et al., 1987), and cutaneous infections (**Table 1**). Here, we describe a case of soft tissue infection by *L. longbeachae* in the index finger, pricked by rose thorns during gardening, in an immunocompetent person. We also review the available literature on these rare soft tissue infections caused by different *Legionella* spp.

**Table 1:** Patient group characteristics.

Reference	Species	Age	Sex	Country	Medical history	Immunosuppression	Exposure	Skin manifestation	Pulmonary involvement	Diagnostic test	Treatment	Outcome
Current case	*L. longbeachae*	61	F	SWE	HTN	No	Rose thorn	Tendovaginitis, tendon sheath necrosis	No	PCR/Seq, Leg-PCR, culture	Levofloxacin	Cured
Desgranges et al. (2020)	*L. longbeachae*	78	F	CH	Temporal arteritis	Yes	Rose thorn, manure	Purulent material in all tendon sheets and CT	No	PCR/Seq, culture	Levofloxacin	Cured
Grimstead et al. (2015)	*L. longbeachae*	70	F	UK	ITP	Yes	Potted plant	Three tender erythematous nodules forearm. Non-purulent	No	PCR/Seq	Ciprofloxacin, azithromycin, rifampicin	Cured
Mentula et al. (2014)	*L. longbeachae*	80	F	FI	AF, HTN PMR	Yes	Broken flowerpot	Swollen hand. Purulent arthritis MCPJ III	No	PCR/Seq, culture	Levofloxacin	Cured
Chou et al. (2016)	*L. longbeachae*	69	F	AUS	HTN	No	Unknown. Florist	Swollen hand, tenosynovitis	No	Culture	Ciprofloxacin	Cured
Qin X et al (Qin et al., 2002)	*L. micdadei*	9	F	US	Healthy	No	Unknown	Enlarged neck mass	No	PCR/Seq	Clarithromycin	Cured
Kilborn et al. (1992)	*L. micdadei*	39	F	US	Renal transplantation	Yes	Unknown	Swollen arm, erythema	Yes	DFA, culture	Erythromycin, rifampin	Amputation/cured
Ampel et al. (1985)	*L. micdadei*	62	F	US	GN, necrotizing vasculitis	Yes	Unknown	Cutaneous abscess leg	Yes	DFA, culture	Cefotaxime, erythromycin	Cured
Valve et al. (2010)	*L. pneumophila*	48	M	FI	Liver cancer, liver transplantation	Yes	Unknown	Infected pyoderma gangrenosum leg	Yes	IFA, culture	Moxifloxacin, fluconazole	Cured
Han et al. (2010)	*L. pneumophila*	65	F	US	ILD and ITPP	Yes	Unknown	Cellulitis leg	No	DFA, culture	Azithromycin, levofloxacin	Death
Brabender et al. (1983)	*L. pneumophila*	71	M	US	Hip replacement COPD. patent ductus arteriosus.	No	Bath in Hubbard-tank	Swollen hip, erythema	No	DFA, culture	Erythromycin lactobionate	Cured
Arnow et al. (1983)	*L. pneumophila*	46	F	US	DPGN	Yes	Enemas	Perirectal abscess	Yes	Culture	Erythromycin, rifampicin	Cured
Waldor et al. (1993)	*L. pneumophila*	66	M	US	Follicular lymphoma	Yes	Unknown	Cellulitis chest	Yes	UAT, DFA	Erythromycin	Cured
Padrnos et al. (2014)	*L. pneumophila*	27	F	US	Pre-B cell LL, Stem cell transplant	Yes	Unknown	Erythematous subcutaneous masses on the leg	Yes	Culture	Azithromycin	Death
Neiderud et al. (2013)	*L. bozemanii*	82	M	SWE	Healthy	Yes	Unknown	Open wound hand	Yes	PCR/Seq, Leg-PCR, serology	Moxifloxacin clarithromycin	Cured
Chee et al (Chee and Baddour, 2007)	*L. maceachernii*	68	F	US	PMR	Yes	Garden work	Swelling, erythema, nodules hand/arm	No	Culture	Levofloxacin	Cured
Loridant et al. (2011)	*L. feeleii*	66	F	FRA	CLL	Yes	Insect/spider bite	Papular lesion, cellulitis, and abscess leg	No	Microscopy, PCR/Seq, culture	Levofloxacin	Cured
Gubler et al. (2001)	*L. cincinnatiensis*	73	F	CH	HTN, nephrotic syndrome, IgA gammopathy	Yes	Unknown	Abscess in jaw, wrist, and arm	No	Microscopy, PCR/Seq, culture	Clarithromycin, rifampin	Cured

Baseline characteristics of patients suffering cutaneous infection caused by a Legionella spp.

F, female; M, male; HTN, hypertension; ITP, immune thrombocytopenic purpura; CT, carpal tunnel; GN, glomerular nephritis; ILD, interstitial lung disease; ITPP, idiopathic thrombocytopenic purpura; DPGN, idiopathic diffuse proliferative glomerulonephritis; LL, lymphocytic leukemia; PMR, polymyalgia rheumatica; DM1, diabetes type 1; DM2, diabetes type 2; COPD, chronic obstructive pulmonary disease; AF, atrial fibrillation; CLL, chronic lymphocytic leukemia; IgA, immunoglobulin A; PIPJ, proximal phalangeal joint; MCPJ, metacarpal phalangeal joint; SWE, Sweden; CH, Switzerland; FRA, France; FI, Finland; US, United States; UK, United Kingdom; CHN, China; AUS, Australia; PCR, polymerase chain reaction; Seq, sequencing; UAD, urinary antigen test; DFA, direct fluorescent staining; IFA, immunofluorescent assay MONOFLUO; Leg-PCR, specific Legionella PCR.

## Case report

A 61-year-old woman, without any history of immunosuppression or severe underlying disease, but treated for hypertension and hyperlipidemia, presented at our hospital with a 1-day history of fever and a painful swollen right index finger, which had started in the tip of the finger. The symptoms had spread to the base of the index finger, also involving her thumb and long finger upon arrival at the hospital. Two days earlier, she had been working in her garden, planting roses with locally purchased bagged soil and groundcover bark. Despite wearing gardening gloves, she had pricked her finger on the rose thorns.

On examination, she reported extreme pain on palpation along the volar aspect of the finger and on attempting extension and flexion of the finger. The index finger showed fusiform swelling with mild redness and warmth as well as some swelling of the thumb and long finger without pain. She had a fever (39.5°C) and chills, while other hemodynamic parameters were stable. She had no respiratory symptoms. White blood cell count was normal, 7.7 × 10^9^ cells/L (normal range 4.3–10.8), and C-reactive protein was 23 mg/L (normal < 5 mg/L).

**Figure 1:**
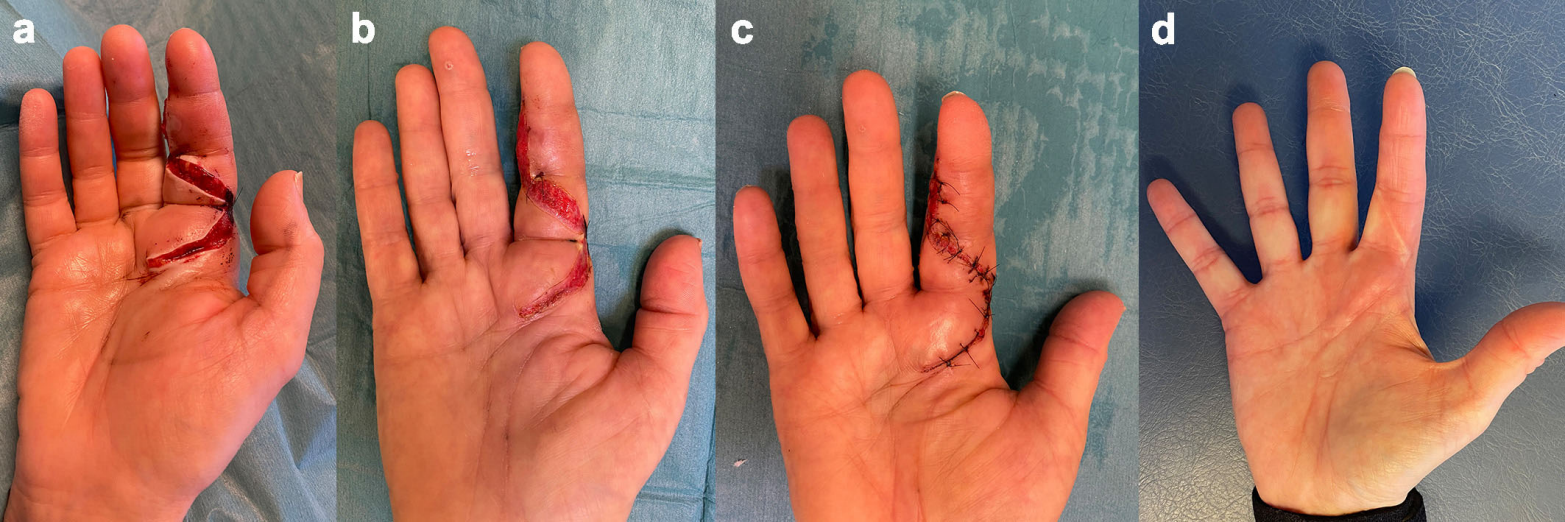
**(A)** Patients’ right hand and index finger at day 1 postoperatively, **(B)** at day 10 postoperatively, **(C)** at day 13 postoperatively, and **(D)** at 2 months postoperatively.

**Figure 2:**
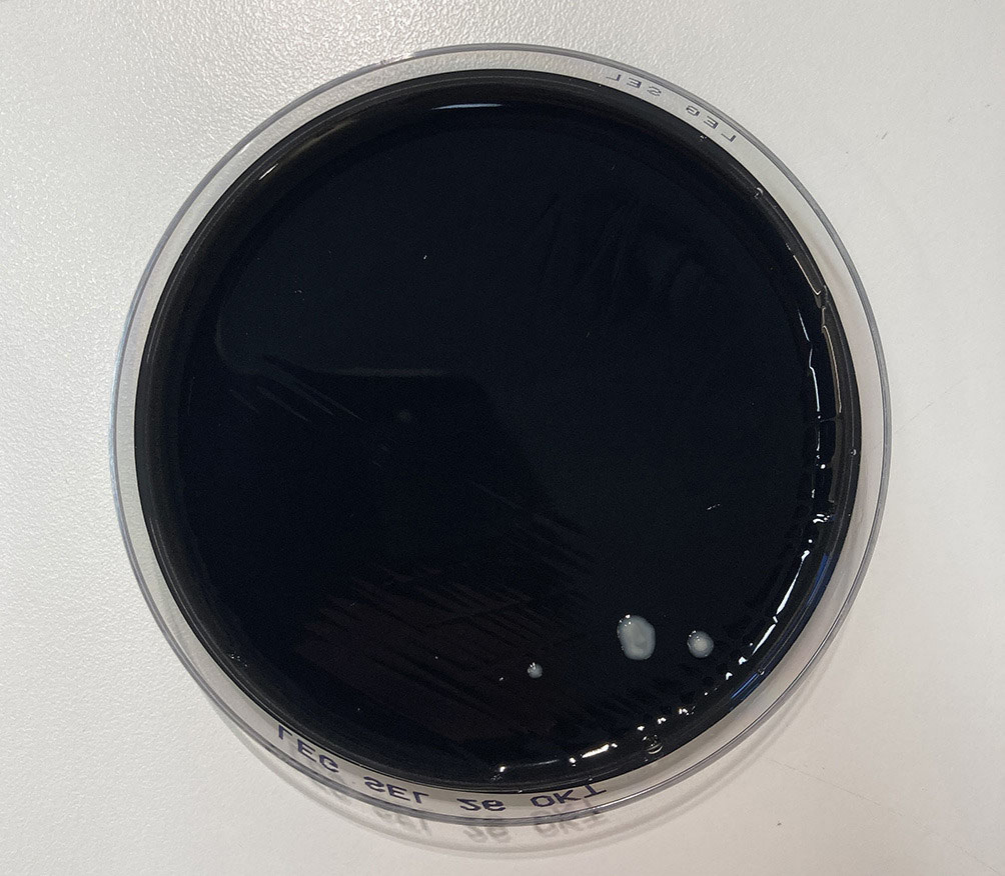
A total of five colonies of *Legionella longbeachae* colonies grew on buffered charcoal yeast extract agar (BCYE).

The patient was promptly taken to operation due to strong suspicion of an aggressive infection. An incision at the level of A1 pulley of the tendon sheath at the base of the index finger showed destruction of the tendon sheath that had dissolved, leaving only brown jellylike remains. The subcutaneous tissue was very swollen and distended from the wound. The incision was extended to the tip of the finger, revealing the same destruction in the rest of the tendon sheath from the A1 to the A5 pulleys. Interestingly, the flexor tendons seemed unaffected as well as the dermis and epidermis, which showed no discoloration. The surgical wound was washed extensively with sterile saline after retrieving tissue culture from the destructed tendon sheath. The tissue was divided into two ESwab tubes (Copan, Brescia, Italy) for microbiological cultivation and 16S rRNA PCR analysis due to the unusual presentation and sent for analysis at the Regional Clinical Microbiology Laboratory in Lund, Sweden. The patient was treated with benzylpenicillin 3 g and clindamycin 600 mg intravenously three times daily. Postoperatively, the patient’s health status remained stable, and her fever dropped, but with remaining subcutaneous swelling distending from the wound and with extensive transparent yellowish secretion (**Figure 1A**). Aerobic standard cultivation was performed with the first sample on blood agar plates (Neogen, Lansing, MI, USA), hematin agar plates (Oxoid Ltd., Basingstoke, UK), Gc-D Agar plates (Difco GC agar base, BD, Franklin Lakes, USA, supplemented with Vitox, Oxoid Ltd., Basingstoke, UK) in 5% CO_2_, and anaerobic cultivation on FAA agar plates and FAA broth (Neogen, Lansing, MI, USA). From the second sample, nucleic acid was extracted and analyzed with 16S rRNA PCR and Sanger sequencing, using forward primer (P5f) 5′-TGC CAG CMG CCG CGG TWA-3′ and reverse primer (P1067r) 5′-AAC ATY TCA CRA CAC GAG CT-3′ (Eurofins Genomics, Ebersberg, Germany) adapted from Relman et al. (1993). The sequencing results from the forward and reverse strands were aligned using BioNumerics software (Applied Maths NV (bioMérieux), Sint-Martens-Latem, Belgium) and analyzed using BLASTn (Bethesda, 2004) that identified *L. longbeachae* with 99% identity (552/553 bases) on postoperative day 3. Based on the PCR results, the treatment regime was changed to levofloxacin 500 mg twice daily with prolonged treatment with benzylpenicillin while waiting on the standard cultures. The *L. longbeachae* result could be confirmed (Cq of 25) using a newly established *in-house* multiplex real-time PCR method, modified from Cross et al. (2016), that targets the 23S–5S rRNA spacer region common to all *L.* spp. and identifies *L. longbeachae*, *L. micdadei*, *L. bozemanii*, and *L. anisa* using species-specific probes (Eurofins Genomics, Ebersberg, Germany). To confirm the PCR result, cultivation was performed with a small amount of remaining sample on buffered charcoal yeast extract agar containing *Legionella* BCYE Growth Supplement (BCYE agar) and BCYE agar containing *Legionella* BMPA Growth Supplement (BMPA agar) (Oxoid Ltd., Basingstoke, UK) and incubated at 35°C and 5% CO_2_. Postoperatively at day 7, a total of five colonies had appeared (**Figure 2**) that could be identified as *L. longbeachae* using matrix-assisted laser desorption/ionization–time of flight (MALDI-TOF) (Vitek MS; bioMérieux, France). The standard cultures showed no bacterial growth. The patient improved by treatment with levofloxacin and was dismissed from the hospital on postoperative day 11. Two weeks after surgery, the patient started physical therapy. The patient stopped levofloxacin treatment after a total of 13 days. The wound then healed, and the swelling improved (**Figures 1B, C**), but the patient developed a painful Achilles tendonitis bilateral as well as generalized muscle pain, which is a well-known side effect of levofloxacin. Treatment with levofloxacin was stopped, even though no previous experience of fewer than 3 weeks of antibiotic treatment has been described among patients with *Legionella* wound infection. The patient started physical therapy and regained full range of motion without bowstringing and normal sensation 2 months postoperatively (**Figure 1D**).

Infection with *L. longbeachae* is a notifiable disease according to Swedish law. Samples were taken from the patient’s garden and water, but *Legionella* cultures were negative. Samples from the patient’s garden gloves were also analyzed with *Legionella* 23S-5S real-time PCR, which showed weak amplification of *Legionella* species at Cq > 35, but no *Legionella* colonies could be cultivated on BCYE agar (i.e., 2 months after the infection).

## Review of reported cases

### Methods

A literature search on PubMed from start through December 2022 using the terms “Legionella”, “Legionella” combined with “longbeachae”, “pneumophila”, “bozemanii”, and “micdadei” with an additional search of combined “soft tissue”, “skin”, “cutaneous manifestation”, “infection”, and “wound”. The retrieved articles were reviewed to identify additional cases of *Legionella* skin and soft tissue infection. The inclusion criteria were cutaneous legionellosis confirmed with PCR or culture positive for *L. longbeachae*, *L. pneumophila*, *L. bozemanii*, *L. feeleii*, *L. micdadei*, or *L. cincinnatiensis*. Exclusion criteria were postoperative infection, septic arthritis, suspected blood-borne infection, or pulmonary involvement preceding the cutaneous infection.

## Results

### Baseline characteristics

Our review included 18 cases of confirmed *Legionella* spp. cutaneous infections. Of those, 5/18 were *L. longbeachae*, 6/18 were *L. pneumophila*, 3/18 were *L. micdadei*, 1/18 was *L. bozemanii*, 1/18 was *L. maceachernii*, 1/18 was *L. feeleii*, and 1/18 was *L. cincinnatiensis*.

Women suffered more commonly a cutaneous infection with *Legionella* spp. than men (14/18, 78%), where the median age was 66 [48–71 interquartile range] (**Table 1**). Country of origin was most commonly the United States (9/18) followed by Finland (2/18), Sweden (2/18), Switzerland (2/18), France (1/18), United Kingdom (1/18), and Australia (1/18).

#### Medical history and immunosuppression

Of the patients diagnosed with a cutaneous infection caused by a *Legionella* spp., 14/18 (78%) were treated with immunosuppressive medication for underlying conditions. These conditions included temporal arteritis, immune thrombocytopenic purpura, polymyalgia rheumatica (PMR), glomerulonephritis, renal transplantation, liver transplantation, interstitial lung disease, lymphoma, and leukemia.

#### Exposure and cutaneous manifestation

All the *L. longbeachae* cutaneous infections manifested after garden work with flowers or flowerpots. This also included the infection related to *L. maceachernii*. For the rest of the infections, the exposure was either unknown or unclear.

#### Diagnostic studies

The most common test to first yield *Legionella* spp. was general 16S rRNA PCR and Sanger sequencing 8/18 (44%) (depicted as PCR/Seq in **Table 1**) followed by culturing on BCYE agar. In addition to 16S rRNA PCR, two of the articles performed a confirmatory *Legionella*-specific PCR that identified *L. bozemanii* (Neiderud et al., 2013) and *L. longbeachae* (current case). Other methods used to detect *Legionella* included direct fluorescent antibody (DFA) assays (Brabender et al., 1983; Ampel et al., 1985; Kilborn et al., 1992; Waldor et al., 1993; Han et al., 2010), immunofluorescent assay MONOFLUO (IFA) (Valve et al., 2010), and cultivation on chocolate agar (Arnow et al., 1983). Urinary Legionella Antigen Test (UAT) was used in two of the cases, but both yielded negative results (Padrnos et al., 2014; Grimstead et al., 2015).

#### Treatment and outcome

All the patients were treated with fluoroquinolones and/or macrolides in single or combination therapy after diagnosis of a *Legionella* spp. Single therapy was prescribed in 9/18 cases (50%). Of those, 6/18 (33%) received fluoroquinolones [levofloxacin 5/6 (83%) (Chee and Baddour, 2007; Loridant et al., 2011; Mentula et al., 2014; Desgranges et al., 2020) and ciprofloxacin 1/6 (17%) (Chou et al., 2016)], and 3/18 (15%) received macrolides [clarithromycin 1/3 (33%) (Qin et al., 2002), erythromycin 1/3 (33%) (Waldor et al., 1993), and azithromycin 1/3 (33%) (Padrnos et al., 2014)]. Combination therapy of fluoroquinolones and macrolides was prescribed in 3/18 (15%) (Han et al., 2010; Neiderud et al., 2013; Grimstead et al., 2015). Of the remaining cases, 5/18 (28%) were treated with macrolides in combination with other antibiotics (Arnow et al., 1983; Brabender et al., 1983; Ampel et al., 1985; Kilborn et al., 1992; Gubler et al., 2001), and 1/18 was treated with fluoroquinolones combined with fluconazole (Valve et al., 2010).

The recommendation for the treatment of Legionnaires’ disease is azithromycin or levofloxacin since they are highly effective and have fewer side effects than erythromycin (Phin et al., 2014). To the best of our knowledge, no specific recommendations exist for cutaneous *Legionella* infections.

## Discussion

Cutaneous infections caused by *Legionella* spp. are rare, and to this date, there are only 18 published cases (including the current case), reporting a diagnostically verified infection. In our case, *L. longbeachae* seemed to selectively attack the tendon sheath, causing it to dissolve into brown discolored remains while sparing the tendon. We found only a few descriptions of intraoperative findings in the literature with no similar description as our findings. This leads us to the conclusion that there is a clear need for a more detailed description of intraoperative findings on cases of legionella wound infections for clinical suspicion of *Legionella* spp. to rise early in the treatment process. Interestingly, Jäger et al. (2014), while studying the effect of *L. pneumophila* infection on the lung tissue, showed that *L. pneumophila* adheres to the alveolar lining and primarily infects alveolar macrophages, causing damage to the alveolar septa. Nevertheless, we found no connection in the literature between the selectiveness of *L. longbeachae* to the tendon sheath and its destruction.

After less than 2 weeks of treatment, the present patient experienced quite severe side effects from the levofloxacin in the form of muscle, joint, and tendon pain. This is a well-known side effect where the risk increases with age over 60 years, concomitant corticosteroid therapy, renal dysfunction, and a history of organ transplantation (Bidell and Lodise, 2016). The present patient had therefore a relatively short levofloxacin treatment compared to other published cases yet regained full recovery. This might help in determining treatment lengths for future patients experiencing similar side effects.

In our review, 78% of the patients were women with a median age of 66 years. This is an interesting finding since epidemiological studies have shown that women are at less risk than men when it comes to developing most infectious diseases. The sex hormone estradiol appears to confer protective immunity, a hormone that is reduced after menopause. This could yet explain the high percentage of women found to suffer from *L. longbeachae* wound infection since the larger part of the women included in our review can be assumed to have been postmenopausal (Gay et al., 2021). Another factor is occupation and lifestyle activities that can play a major role in exposure to pathogens. Still, the authors found no published studies indicating that gardening was more common among women than men. Of the 18 included patients in our review, 78% were treated with immunosuppressive medication for underlying conditions. Nevertheless, in our case, the patients did not have any history of immunosuppression. In otherwise healthy patients, it is uncommon to have complex rare skin infections, which might play a role in the choice of microbial analyses. To enable the detection of *Legionella*, the clinician must specifically ask for that analysis since *Legionella* requires specific growth conditions, or the use of 16S rRNA analysis was the most common test to first yield *Legionella* spp. in the reviewed cases including the present case. In our clinic, it is not standard to order a wide range of microbial cultures when an otherwise healthy patient presents with septic tenosynovitis. Again, a culture-negative infection that is hard to treat with broad-spectrum antibiotics appears, needing repeated surgery with surgical debridement and washing before the infection subsides without a clear diagnosis. One might wonder if the number of immunosuppressed patients in these cases is perhaps not representative of the total population but rather the population that is prescribed the right cultures due to a higher risk for complicated infections. This means that the number of true cases is hard to estimate. In our region, we are only aware of three additional cutaneous infections identified by 16S rRNA analysis, two of which could be cultivated (*L. sainthelensis* and *L. pneumophila*).

An obstacle to suggesting that one expands the prescribed microbial analysis per patient is most certainly the cost, but also time. An additional 16S rRNA PCR is time-consuming and expensive and, most importantly requires a sample collected from a normally sterile location. Otherwise, it is impossible to interpret the result correctly since the PCR will amplify all the bacteria present in the sample. Therefore, a clear protocol is needed to guide physicians and surgeons in their choice of microbial analyses.

All the reviewed *L. longbeachae* cutaneous infections (5/18) manifested after garden work with flowers or flowerpots. A recent study on the frequency of *L. longbeachae* on gardening gloves using a *L. longbeachae*-specific PCR showed the presence of the bacterium on 14% of the gloves (11/76), postulating that gloves could be a vector and contribute to *Legionella* infections (Chambers et al., 2021). In our case, 23S–5S rRNA *Legionella*-specific real-time PCR on a swab sample taken from the patients’ gloves, stored for 2 months since the debut of infection in a dry place, gave a low amplification for PAN-*Legionella* but not specifically for *L. longbeachae*. Cultivation on BCYE and BMPA media did not result in *Legionella* growth, whereby the source of the bacteria could not be determined. To identify the source of the *Legionella* spp. causing the infection is time-consuming work involving sampling and cultivation of many environmental samples followed by molecular typing to determine if there is an epidemiological match to the patient strain. For various types of water reservoirs, the sampling methods are rather well established, and the presence of *Legionella* spp. has been well studied (Kanarek et al., 2022). For soil, it is more problematic and varies depending on the country. In a soil survey conducted in Australia in 1989–1990, 73% of potting soil samples tested positive for *Legionella*, and 75% of these contained *L. longbeachae*. In a study conducted in Switzerland in 2009, 21/46 (46%) of potting soils contained *Legionella* spp (Steele et al., 1990; Casati et al., 2009). The discrepancies between countries might be explained by a variety of factors, including climate, the composition of the soil in the bags, and the methods used in production. During a *L. longbeachae* outbreak in Sweden in 2018, Löf et al. (2021) tried to identify the disease-causing strains by sequencing isolated patient and environmental strains. They could determine a higher risk for *L. longbeachae* infection if the patient had been handling bagged compost and/or soil, but a direct connection to the patient isolates could not be established by next-generation sequencing (NGS) since the soil isolates were polyclonal, thus containing several *L. longbeachae* strains (Löf et al., 2021).

## Conclusion

To the best of our knowledge, this is the largest review of cutaneous *Legionella* cases, and our case adds information to the limited existing literature on similar patients. *Legionella* spp. may be an underdiagnosed source of extrapulmonary infections due to the requirement for specific media and diagnostic methods. These infections can go undetected for a long time and cause serious infections. We believe special attention and heightened awareness of these infections during history taking and clinical examination of patients presenting with atypical cutaneous infections is therefore important.

## Ethics statement

Written informed consent was obtained from the individual for the publication of any potentially identifiable images or data included in this article.”

## Author contributions

All authors contributed to the article and approved the submitted version.
